# Rate of tarsal and metatarsal bone mineral density change in adults with diabetes mellitus and peripheral neuropathy: a longitudinal study

**DOI:** 10.1186/s13047-023-00606-2

**Published:** 2023-02-13

**Authors:** Nicholas J. Youmans, Rachana S. Vaidya, Ling Chen, Hyo-Jung Jeong, Alexa York, Paul K. Commean, Mary K. Hastings, Jennifer A. Zellers

**Affiliations:** 1 Washington University School of Medicine, MSC 8502-66-1101, 4444 Forest Park Avenue, St. Louis, MO 63108 USA; 2 Marquette University, Milwaukee, WI USA; 3 University of Wisconsin-Milwaukee, Milwaukee, WI USA

**Keywords:** Foot, Ankle, Orthopedics, Endocrinology, Computed tomography, Calcaneus, Bone loss

## Abstract

**Background:**

In people with diabetes (DM) and peripheral neuropathy (PN), loss of bone mineral density (BMD) in the tarsals and metatarsals contribute to foot complications; however, changes in BMD of the calcaneal bone is most commonly reported. This study reports rate of change in BMD of all the individual bones in the foot, in participants with DM and PN. Our aim was to investigate whether the rate of BMD change is similar across all the bones of the foot.

**Methods:**

Participants with DM and PN (*n* = 60) were included in this longitudinal cohort study. Rate of BMD change of individual bones was monitored using computed tomography at baseline and 6 months, 18 months, and 3–4 years from baseline. Personal factors (age, sex, medication use, step count, sedentary time, and PN severity) were assessed. A random coefficient model estimated rate of change of BMD in all bones and Pearson correlation tested relationships between personal factor variables and rate of BMD change.

**Results:**

Mean and calcaneal BMD decreased over the study period (*p* < 0.05). Individual tarsal and metatarsal bones present a range of rate of BMD change (-0.3 to -0.9%/year) but were not significantly different than calcaneal BMD change. Only age showed significant correlation with BMD and rate of BMD change.

**Conclusion:**

The rate of BMD change did not significantly differ across different foot bones at the group level in people with DM and PN without foot deformity. Asymmetric BMD loss between individual bones of the foot and aging may be indicators of pathologic changes and require further investigation.

**Trial registration:**

Metatarsal Phalangeal Joint Deformity Progression—R01. Registered 25 November 2015, https://clinicaltrials.gov/ct2/show/NCT02616263

## Introduction

Diabetes mellitus (DM) is a metabolic disorder that affects an estimated 463 million people worldwide [1]. In 2021, approximately 45% of adults with DM worldwide remained undiagnosed and, consequently, medically unmanaged [2]. Poorly controlled DM causes damage to many different body systems and presents with a complex clinical presentation [3]. Peripheral neuropathy (PN) occurs in nearly 50% of adults with DM and results in the loss of protective sensation and motor function [4, 5]. The distal to proximal progression of PN in DM results in foot complications that are common and costly [6]. In particular, foot deformity and fracture are key contributors to the events leading to ulceration, infection, and amputation [3, 7–10] and people with DM have a 25 times greater risk of lower limb amputation than those without DM [11]. Thus, identifying modifiable factors contributing to the development of foot deformity is critical for successfully managing DM and preventing serious PN consequences, such as ulcers and amputation [12–14].

Foot fracture risk and deformity are associated DM complications [15] and have been linked to the loss of tarsal and metatarsal bone mineral density (BMD) [16, 17]. Of particular interest, individuals with type 2 DM showed the most significant elevation of fracture risk in the foot, where the relative risk was 37% higher than that in the control population without type 2 DM, suggesting the involvement of PN in fracture predisposition [18]. BMD is a strong predictor of fracture and adults with DM have been observed to have higher BMD but lose BMD at a faster rate than age-matched controls [8, 19, 20]. Due to dual-energy X-ray absorptiometry scanning protocols, most studies of foot BMD in DM have measured only the calcaneus [18, 19, 21–25]. However, any foot bone or joint can be the site of DM-associated foot deformity or fracture, and calcaneal BMD alone may not reflect what is occurring across distal foot bones and joints. Volumetric quantitative computed tomography has allowed quantification of individual foot bone BMD, however, the studies that have been completed with this imaging technique are cross-sectional in study design [26, 27]. A recent cross-sectional study, for example, demonstrated differences in BMD between the talus and calcaneus in healthy individuals, yet whether the bones of the foot lose BMD similarly over time is unknown. Our study addresses this gap by measuring longitudinal changes in BMD of individual tarsals and metatarsals in people with DM and PN.

This study aims to identify the rate of BMD change in the tarsals and metatarsals of adults with DM and PN. We hypothesized that BMD would decrease over time in all bones. Our primary null hypothesis was that there would be no significant differences in rate of BMD change between the tarsal and metatarsals compared to the calcaneus. We further hypothesized that factors such as age, sex, activity level, and severity of PN would affect the rate of BMD change. Considering altered bone turnover and concurrent risk for increased secondary complications of DM, the rate of BMD change in the foot may provide clues to improve screening and management of long-term complications of the disease, reduce healthcare costs, and improve the quality of life for adults affected by DM.

## Methods

### Study design

This is a prospective, longitudinal clinical trial of secondary outcomes included in a larger clinical trial (ClinicalTrials.gov: NCT02616263). Participants with type 2 DM and PN were randomized in one of two intervention cohorts, which received targeted strengthening and stretching interventions on either the foot or shoulder. For the purposes of this analysis, all participants were assessed as one cohort. A sensitivity analysis was completed to ensure there were no significant differences in BMD between the intervention groups prior to collapsing the groups. The detailed findings of the sensitivity analysis are reported in the results section. Data were collected over the course of three to four years at four-time points: baseline (T1), 0.5 years (T2), 1.5 years (T3), and at the conclusion of the study (3–4 years after baseline, T4) [28, 29]. Time was treated as a continuous variable to account for variation in the actual date of reassessment for each participant.

### Participants

Participants were recruited from the Recruitment Enhancement Core of the Institute of Clinical and Translational Sciences at the Washington University School of Medicine in St. Louis, email blasts to a senior center, along with several databases (e.g., Research Participant Registry through Volunteers for Health, and patient databases of the Applied Biomechanics and Human Biodynamics Laboratories). Inclusion criteria were type 2 DM diagnosed by the participants’ healthcare providers and PN assessed by the study team. In order to capture the multi-system effects of PN, presence of PN was defined as any of the following: inability to feel a 5.07 Semmes Weinstein Monofilament in at least one location on the plantar aspect of the foot, a vibration perception threshold greater than 25 V on biothesiometry, or a score of greater than or equal to 2 on the Michigan Neuropathy Screening Instrument (1 point is given for each foot abnormality including deformities, dry skin, calluses, infections, fissures, and ulcers) [30]. For the purposes of analysis, PN severity was represented by each participant’s Michigan Neuropathy Screening Instrument score. Exclusion criteria were PN of non-DM etiology, ankle brachial index of < 0.9 or > 1.3 indicating severe arterial disease [11, 31], amputation of more than one toe, acute shoulder injury, pregnancy, > 180 kg—the weight limit capacity of the magnetic resonance imaging (part of the parent study) scanner, active neuropathic ulceration, inability to walk without a device or complete required testing, presence of non-magnetic resonance imaging compatible metal implants, age greater than 75 years, or completion of less than two computed tomography (CT) scans over the course of the study. Race was self-reported on intake. Each participant completed the Foot and Ankle Ability Measure [32] for descriptive purposes. The Foot and Ankle Ability Measure is a self-reported measure of foot and ankle specific symptoms and function, with higher scores indicating less disability/symptoms (maximum score of 100). No participants experienced non-healing plantar ulceration or amputation during the course of the study. While exclusion criteria included history of non-healing ulceration, participants were not withdrawn from the study if they acquired wounds during the study. Nine participants self-reported occurrence of foot wounds (from non-traumatic causes as well as known trauma – cuts, scratches, etc.) during the course of the study, which were able to heal. Wounds were defined as any break in skin continuity of any depth. All wounds reported by participants were non-tunneling. Wound management for all instances of foot wounds required neither immobilization nor offloading. The participant demographics and characteristics are in Table 1.

**Table 1:** Participant Demographics and Characteristics

Characteristic	Number of Participants	Mean	SD
Sex	34 F/26 M	-	-
Age at baseline (years)	60	67	6
Race	16 Black or African-American/ 42 White/ 3 More than one race	-	-
Height (cm)	60	169	9
Weight (kg)	60	99.6	20.7
Body Mass Index (kg/m^2^)	60	35.1	7.4
Hemoglobin A1C (%)	59	7.1	1.3
Michigan Neuropathy Screening Instrument Score	60	5	1
Foot and Ankle Ability Measure Total	60	80	20
Diabetes duration at baseline (years)	60	14	10

*SD* Standard Deviation

### Bone mineral density acquisition

**Fig. 1:**
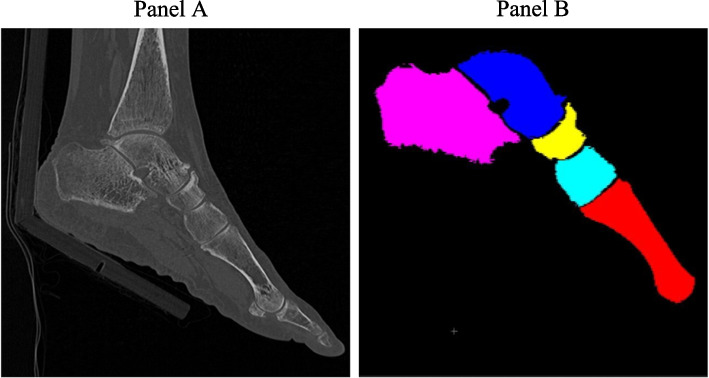
Images of Foot Positioning for CT scan and Segmentation of foot bones. Panel **A** CT scan of target leg resting on a stabilization board holding the ankle in 30 degrees of plantar flexion and metatarsophalangeal joints in the resting position; Panel **B** Sagittal cross-section of segmentation of tarsals and metatarsals (from left: calcaneus, talus, navicular, 1^st^ cuneiform, 1^st^ metatarsal)

A spiral CT scanner (Siemens Biograph 40 TruePoint Tomograph) was used to scan each participant’s lower extremity capturing the entire ankle mortise of the selected side as well as all tarsals and metatarsals. Scans were performed at all four time points. Participants underwent CT scans of the foot in 30 degrees of plantarflexion and metatarsophalangeal joints in resting position (Fig. 1: Panel A). A CT calibration phantom (Mindways, Austin, TX, USA; Calibration value: L11G 11F1 1171 611L) containing multiple discs of calcium hydroxyapatite of different densities was placed in front of the foot for all scans. The CT scanning parameters were 0.5 s rotation time, 64 × 0.6 mm collimation, 220 mAs, 120 kVp, a pitch of 1, a 512 × 512 matrix, and a voxel size of 0.6 × 0.6 × 0.6 mm^3^.

BMD of each of the 12 tarsals and metatarsals was obtained using a previously published protocol [17, 27]. Each tarsal and metatarsal was individually segmented, and Hounsfield Unit (HU) was converted to BMD (mg/cm^3^) using the calibration phantom (Fig. 1: Panel B).

### Participant activity level

The physical activity level of each participant was obtained at baseline (T1) using an ActiGraph wGT3X-BT activity monitor (ActiGraph, Pensacola, FL). Participants were asked to wear the ActiGraph full-time for 5 days, of which the middle 3 days were used for analysis (to ensure only days with a full 24-h wear time were included in analysis). The ActiGraph was placed on the participant’s wrist of the non-dominant hand using a band that prevented removal during the 5-day collection period. Daily step count and sedentary time (minutes) were calculated using ActiLife software and were included in statistical analysis.

### Medication and diabetes management

Participants’ medication use and diabetes management strategies were obtained via self-report at baseline (T1). Medications were classified as statins or non-statins for use in statistical analysis because statin use is known to increase BMD [33]. No participants reported taking bisphosphonate medications, a common class of medications used to treat osteoporosis [34]. The diabetes management strategies were collected with four choices (i.e., diet, exercise, oral medications, and insulin) and allowed multiple selections.

### Statistical analysis

All BMD measures were checked for normality assumptions via Shapiro–Wilk test and visual inspection of QQ plots and histograms. All data met normality assumptions. Alpha level of significance was set at 0.05 a priori for all analyses.

A random coefficients model (linear mixed model) was used to estimate the annual rate of change (slope) of BMD (mg/cm^3^ per year) in the calcaneus and mean of all tarsal and metatarsal bones. This model allowed both intercept and slope to vary randomly between subjects and fitted a separate regression line for each individual subject. A separate random coefficients model analysis was performed to predict annual rate of BMD change for individual tarsals and metatarsals.

To determine if there are any significant differences in rate of BMD change between the bones of the foot, an additional random coefficients model was built to test for significance of bone name by time interaction with significant interaction meaning a different rate of BMD change between bones. In the current body of literature, the calcaneus is the most frequently used bone to assess foot BMD [19, 35], so the calcaneus was selected as the referent value for this analysis. The estimated rate of BMD change of each individual tarsal and metatarsal are reported.

Personal factor variables that were hypothesized to affect mean BMD (mg/cm^3^) and calcaneal BMD (mg/cm^3^) included age, sex, step count, sedentary time, and Michigan Neuropathy Screening Instrument score. A covariance pattern model (linear mixed model) was fit to assess the association between the personal factors and BMD variables and accounted for the correlation of repeated measures of BMD from the same subject over time. Relationships between personal factor variables and rate of BMD change (mg/cm^3^ per year) were tested using Pearson correlation coefficient.

## Results

Participant characteristics are included in Table 1. Ten participants managed their diabetes with diet, 10 with exercise, 44 with oral medications, and 19 with insulin.

**Fig. 2:**
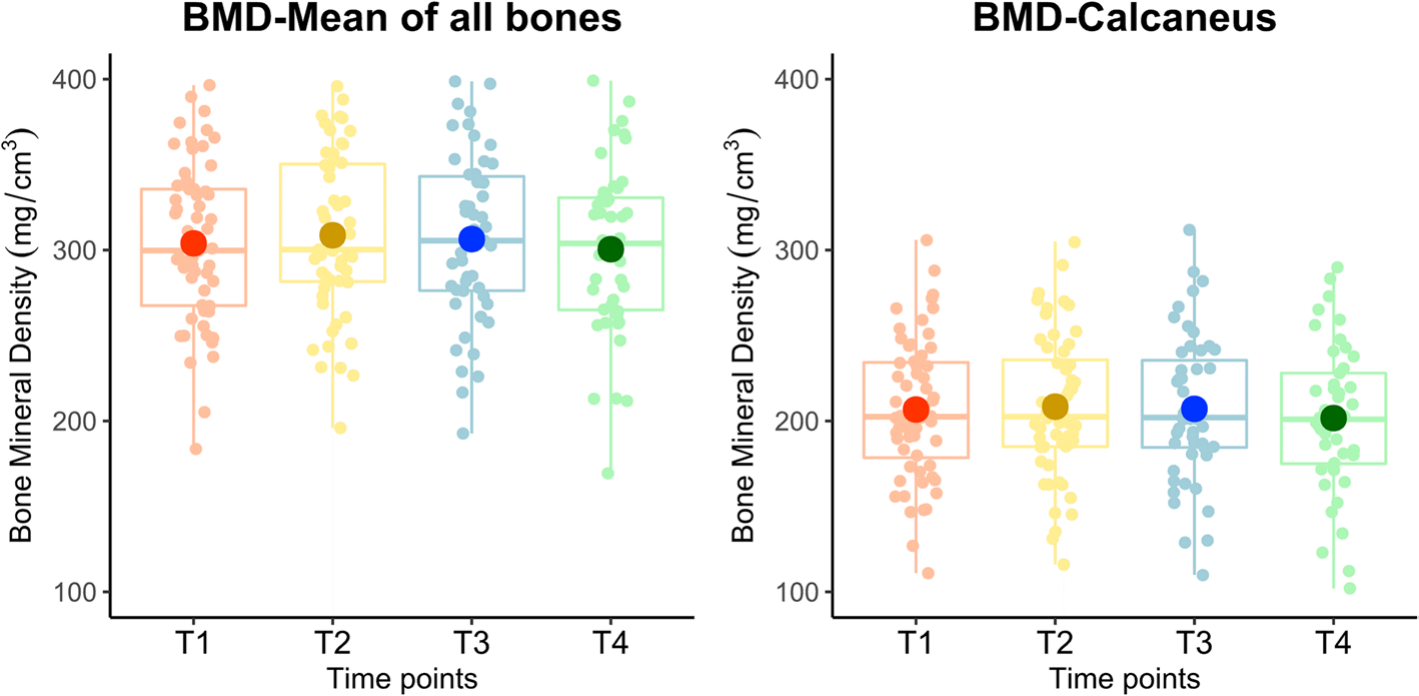
Box plot of (**A**) Bone mineral density (BMD) at all timepoints averaged for all bones, including tarsals and metatarsals and (**B**) for the calcaneus, alone. Bone mineral density (BMD) is in mg/cm^3^. T1: baseline; T2: 0.5 years; T3: 1.5 years; T4: 3–4 years

The mean rate of BMD change of all tarsals and metatarsals (*N* = 60) was -1.57 mg/cm^3^ per year (*p* = *0.0447)* (Fig. 2A). The mean rate of calcaneal BMD (*N* = 60) change was -2.03 mg/cm^3^ per year (*p* = *0.0004)* (Fig. 2B). There was no statistically significant interaction between group assignment (shoulder or foot intervention) and time for both rate of mean BMD change (mg/cm^3^ per year) (*p* = *0.*3207) and rate of calcaneal BMD change (mg/cm^3^ per year) (*p* = *0.1333*) (Table 2), suggesting that the intervention provided in the parent study did not influence the rate of BMD change. Therefore, all participants were combined to form a single cohort for the purposes of this analysis.

**Table 2:** Mean BMD and Calcaneal BMD by Group Assignment

Group Assignment	Number of Participants	Rate of Mean BMD Change^a^ (SD)^b^	Rate of Calcaneal BMD Change^a^ (SD)^b^
Shoulder	31	-1.66 (1.73)	-2.09 (1.62)
Foot	29	-1.46 (1.52)	-1.96 (1.43)

Rate of mean BMD change reflects the averaged value of all tarsal and metatarsal bones

^a^mg/cm^3^ per year

^b^Standard Deviation (SD)

### Individual tarsal and metatarsal bone mineral density

The results of a linear mixed model analysis using each tarsal and metatarsal bone as a single input variable and calcaneal BMD as a reference variable indicate that the rate of BMD change (mg/cm^3^ per year) was not significantly different between the calcaneus and any other bone included in the analysis (*p* = *0.9966*). Descriptive statistics for rate of BMD change (mg/cm^3^ per year) for individual bones are reported in Table 3.

**Table 3:** Rate of BMD Change for Individual Tarsals and Metatarsals (*N* = 60)

Bone	BMD Mean^a^ ± SD	Rate of BMD Change^b^ (SE)	*p*-value	95% CI
1^st^ Metatarsal	298 ± 54	-1.98 (0.79)	0.015*	-3.57, -0.40
2^nd^ Metatarsal	401 ± 66	-1.24 (1.07)	0.259	-3.38, 0.89
3^rd^ Metatarsal	374 ± 60	-1.62 (0.92)	0.082	-3.46, 0.21
4^th^ Metatarsal	365 ± 66	-1.53 (1.04)	0.149	-3.62, 0.57
5^th^ Metatarsal	371 ± 70	-1.23 (0.95)	0.203	-3.14, 0.68
1^st^ Cuneiform	272 ± 53	-1.54 (0.96)	0.115	-3.47, 0.39
2^nd^ Cuneiform	315 ± 51	-1.38 (0.76)	0.074	-2.90, 0.14
3^rd^ Cuneiform	259 ± 47	-0.97 (0.58)	0.103	-2.13, 0.20
Cuboid	202 ± 37	-1.25 (0.57)	0.032*	-2.39, -0.11
Navicular	310 ± 58	-1.46 (0.86)	0.093	-3.18, 0.25
Talus	294 ± 49	-2.54 (0.68)	0.0005*	-3.91, -1.17
Calcaneus	207 ± 41	-2.03 (0.53)	0.0004*	-3.09, -0.96

*CI* Confidence Interval

^a^mg/cm^3^; Standard Deviation (SD)

^b^mg/cm^3^ per year; Standard Error (SE)

### Factors influencing rate of BMD change

The rate of mean BMD change (mg/cm^3^ per year) and rate of calcaneal BMD change (mg/cm^3^ per year) were not impacted by sex, PN severity as determined by score on the Michigan Neuropathy Screening Instrument, daily step count, sedentary time, or statin use (Table 4). Data for daily step count and sedentary time was obtained from ActiGraph data, which was initiated after the beginning of the study, resulting in an incomplete baseline data set for these variables. There was a significant main effect of age on mean (*p* = *0.0086*) and calcaneal (*p* = *0.0256*) BMD (mg/cm^3^) with lower BMD associated with older age. The linear mixed effects model analysis suggests that the average mean BMD decreases by 2.1793 mg/cm^3^ (*p* = *0.0086*) and calcaneal BMD decreases by 2.19 mg/cm^3^ (*p* = *0.0256*) with every one-year increase in age. Rate of change of BMD (mg/cm^3^ per year) was also negatively related to age.

**Table 4:** Effect of Personal Factors on Rate Mean BMD and Calcaneal BMD Change

Personal Factors	Number of Participants	Mean BMD		Calcaneal BMD	
		Pearson correlation coefficient	*p*-value	Pearson correlation coefficient	*p*-value
Sex (F/M)	60	0.043	0.7426	-0.095	0.4723
Michigan Neuropathy Screening Instrument Score	60	-0.158	0.2270	-0.182	0.1633
Age (years)	60	-0.282	0.0288*	-0.322	0.0120*
Daily Step Count	35	-0.115	0.5118	-0.157	0.3689
Sedentary Time (minutes)	35	0.123	0.4799	0.042	0.8088
Statin Use (Y/N)	59	-0.056	0.6747	-0.131	0.3211

*Statistically significant *p*-value; Female (F); Male (M); Yes (Y); No (N)

## Discussion

This is the first study to report longitudinal changes in tarsal and metatarsal BMD in a group of individuals with DM and PN. We observed a decreasing rate of mean BMD (-1.57 mg/cm^3^ per year; -0.5% per year) and calcaneal BMD (-2.03 mg/cm^3^ per year; -1% per year) change over three years (indicating BMD loss), with a range of rates of tarsal and metatarsal BMD change of -0.3% to -0.9% per year. Our study identified that mean BMD (mg/cm^3^) and calcaneal BMD (mg/cm^3^) are lower in older individuals, and the rate of BMD loss is more rapid with advancing age. Previous studies on patients with DM show a rate of calcaneus BMD change in the range of -0.5% per year to -2.5% per year [19, 36]. We observed a comparable rate of BMD change in calcaneus at -1% per year.

A recent study in healthy population shows asymmetric BMD within individual bones of the foot [37], however, the present study is the first to report longitudinal changes in BMD in all of the tarsal and metatarsal bones in patients with DM and PN. We observed similar but not significant differences between rates of BMD change in individual tarsals and metatarsals, with the talus showing the greatest rate of BMD change (-0.9% per year), while the third cuneiform showed the lowest rate of BMD change (-0.3% per year). Our aim was to investigate whether rate of BMD change is similar between the tarsals and metatarsals, compared to the calcaneus, thus each participant in the study served as their own internal control. There was no difference in the rate of BMD change (mg/cm^3^ per year) of the calcaneus compared to any other foot bone, suggesting that the calcaneus could be used as an overall indicator of foot BMD. However, our sample of individuals with DM and PN did not have any occurrences of significant foot complications (i.e., non-healing ulceration, neuropathic Charcot arthropathy or amputation) during the 3–4 years study period, therefore, we are unable to assess the utility of individual tarsal/metatarsal BMD monitoring for identification and prevention of foot complications. A prior study of bone changes in individuals with DM found that asymmetry in BMD changes from the left foot to the right foot was an indicator of Charcot deformity [27, 38]. It may be that asymmetrical BMD loss in individual tarsal and metatarsal bones could serve as an early indicator of loss of foot structural integrity. Future studies to elucidate the utility of screening for BMD changes in individual foot bones would be beneficial.

We also studied the effect of other personal factors on rate of BMD change and found that only age related to BMD and rate of BMD change. Greater PN severity, sex, physical activity, and statin use were not associated with rate of BMD change. Prior studies have reported a more rapid loss of mean BMD in females compared to men, which is counter to our findings [19, 39, 40]. Physical activity and body mass index (BMI) have also been shown to impact rate of BMD loss in a population of similarly aged women to those included in our study (60–80 + years) [41]. We may not see similar effects of physical activity and BMI on rate of BMD change because the range of physical activity and BMI values may not be sufficient to explore this variable in our study. It is also possible that specific elements or methods of activity are more important than volume of activity, such as foot loading pattern, foot intrinsic muscle strength, or type of footwear. The current body of literature on exercise and rate of BMD change supports that body weight exercises in this age population can increase BMD in the hip and lumbar spine [42, 43], but no research has been completed on observing rate of foot BMD change with mild to moderate strength training in this population.

One limitation of our study is the limited severity of foot deformity and dysfunction in this sample of the population of individuals with DM and PN as reflected in absence of current ulceration at intake. More severe BMD changes may be observed in a population with a higher incidence of ulceration, fracture, or amputation. Second, there were missing data and variability in the timing of the 4^th^ time point due to the COVID-19 pandemic, which resulted in a relatively small sample size. We have mitigated this variability in our statistical analysis, which assesses time from baseline as a continuous variable to account for any variation in timing of data collection. Third, current technology and methodology makes manual segmentation and BMD calculation for individual bones not viable for translation of our measures into a clinical setting. However, there is potential for future use of artificial intelligence to automate and streamline this process. This study adds to a growing body of literature demonstrating that volumetric quantitative computed tomography as a way of determining BMD for individual tarsals of the foot provides a more comprehensive picture of bone-by-bone health than previous methodologies and can serve as a framework for taking a more fine-tuned approach to monitoring foot bone health. Fourth, to track step count and sedentary time, ActiGraph data collection was added to the study after the study had begun, leading to missing T1 data for the earlier participants. To address this missing data, incomplete ActiGraph data sets were omitted from statistical analysis. Finally, BMD, although indicative of bone quantity, is a fairly blunt measure of bone quality. It is important to note that the annual BMD change reported in our study group does not exceed previously published values for least significant change using this imaging modality [27]. Small changes in BMD may not be detectable with currently available imaging modalities on an individual patient basis. Future work that includes the use of high resolution peripheral quantitative CT, will provide an important contribution to understanding the role of redistribution of BMD and changes in the microarchitecture of the cortical and trabecular bone, important for understanding bone quality and strength.

In summary, we observed individuals with DM and PN to lose BMD at rates comparable to those reported in non-diabetic individuals in the literature. Aging increased the rate of BMD loss. Rate of BMD loss was not significantly different across tarsal/metatarsal bones, and further study is warranted to identify if individuals with asymmetric bone loss in the foot are at risk of negative foot-related outcomes including foot deformity.

## Data Availability

The datasets for the current study are available from the corresponding author on reasonable request.

## Abbreviations

DM: Diabetes Mellitus
BMD: Bone mineral density
CT: Computed tomography
SD: Standard Deviation
PN: Peripheral neuropathy
